# Outcome and safety of single-dose docetaxel–augmented targeted radionuclide therapy using [¹⁷⁷Lu]Lu-PSMA-617 radioligand therapy in treatment-refractory mCRPC: preliminary evidence from a pilot experience

**DOI:** 10.1186/s13550-026-01408-w

**Published:** 2026-03-16

**Authors:** Niklas Ebert, Florian Rosar, Caroline Burgard, Mark Bartholomä, Stephan Maus, Connor Hein, Arne Blickle, Samer Ezziddin, Fadi Khreish

**Affiliations:** 1 Department. of Nuclear Medicine, Saarland University - Medical Center, Homburg, Germany; 2 Department of Nuclear Medicine, Marburg University, Campus Fulda Pacelliallee 4, D-36043 Fulda, Germany

## Abstract

**Background:**

the aim of this study is to investigate the safety profile and efficacy of single-dose docetaxel augmented targeted radionuclide therapy (TRT) in the form of prostate-specific membrane antigen (PSMA)- targeted radioligand therapy (RLT) using Lutetium-177 (177Lu) in patients who have progressed on PSMA-617 RLT. A retrospective analysis was conducted on 10 mCRPC patients (median age: 68 years; range: 51–74 years) who were progressing on prior RLT. The patients received one cycle of treatment involving 50 mg/m² of docetaxel combined with [^177^Lu]Lu-PSMA-617. Efficacy was assessed via biochemical (PSA, PCWG3) and molecular imaging endpoints (TLP/MTV via [68Ga]Ga-PSMA-11 PET/CT). PSA-progression-free survival (PSA-PFS) and overall survival (OS) were analyzed using Kaplan–Meier statistics. Toxicity was graded by CTCAE v5.0.

**Results:**

After single-dose docetaxel augmented [^177^Lu]Lu-PSMA-617 RLT, 60% of the patients showed a disease stabilization for 4–8 weeks based on PSA (10% partial remission and 50% stable disease). Based on the molecular imaging-based response assessment, 62.5% of the patients showed a disease stabilization (25% parietal response and 37.5% stable disease). The median PSA-PFS and OS thereafter were 4.4 months (95%CI: 1.3–7.6 months) and 7.2 months (95%CI: 2.9–11.5 months), respectively. The augmentation therapy was well-tolerated without any serious acute adverse events. During the first 4–8 weeks thereafter 3 patients exhibited transient grade 3 anemia and one patient a transient grade 2 nephrotoxicity.

**Conclusions:**

This pilot experience demonstrates that single-dose docetaxel application to [^177^Lu]Lu-PSMA-617 radioligand therapy is feasible and may present an option for late-stage heavily pretreated mCRPC progressing on PSMA-617 RLT. Formal study of this combination is warranted.

## Introduction

Targeted radionuclide therapy (TRT), including radioligand therapy (RLT) targeting the prostate-specific membrane antigen (PSMA) using PSMA ligands labeled with the beta emitter Lutetium-177 (^177^Lu) has shown excellent results in various studies regarding safety and efficacy [1–4]. Recently, RLT with [^177^Lu]Lu-PSMA-617 has been approved by FDA and EMA as a treatment option for men with late-stage metastatic castration-resistant prostate cancer (mCRPC) [5]. Recently, clinical evidence for PSMA-targeted RLT has been generated in heavily pretreated patient populations who had already exhausted multiple prior lines of systemic therapy, including androgen receptor–directed agents and taxane-based chemotherapy [6]. However, some patients do not respond to this antitumor modality showing primary resistance while others eventually progress after initial response, exhibiting a so-called secondary resistance to [^177^Lu]Lu-PSMA. One hypothetical cause for this observed resistance could be heterogeneity of tumor cells with partial development of reduced sensitivity to beta-radiation, especially in patients undergoing anti-androgen therapies [7]. In such setting intensifying treatment could be considered via combination therapies. For example, combining androgen-receptor signaling inhibitors (ARSI) and RLT, like [^177^Lu]Lu-PSMA-617 with enzalutamide, or employing a tandem therapy strategy by combining alpha and beta radiation, such as [^225^Ac]Ac-PSMA-617 with [^177^Lu]Lu-PSMA-617, may offer promising options [8–10]. Recent evidence suggests that combination therapy strategies, such as tandem protocols combining [²²⁵Ac]Ac-PSMA-617 with [¹⁷⁷Lu]Lu-PSMA-617, may preserve therapeutic efficacy while substantially improving the safety profile, in particular xerostomia [11]. Another approach for patients primarily irresponsive to PSMA-RLT might be the use of a chemotherapeutic agent as radiosensitizer and synergistic enhancer of the RLT antitumor effect.

As accessible research on this topic is scarce, the aim of this study is to investigate the safety profile and efficacy of single-dose docetaxel augmented [^177^Lu]Lu-PSMA-617 RLT in patients who have progressed on PSMA-617 RLT.

## Methods

### Patients and ethics

This retrospective analysis consists of 10 patients with mCRPC who received one cycle of docetaxel (50mg/m^2^) chemotherapy in addition to [^177^Lu]Lu-PSMA-617 RLT after showing disease progression under [^177^Lu]Lu-PSMA-617 RLT (*n* = 8) or [^225^Ac]Ac-PSMA-617 / [^177^Lu]Lu-PSMA-617 tandem RLT (*n* = 2). This retrospective analysis includes patients who showed progressive disease under PSMA-RLT, were clinically and conditionally able to receive docetaxel chemotherapy, and exhibited sufficient PSMA expression on [⁶⁸Ga]Ga-PSMA-11 PET/CT scans. No predefined, quantitative, SUV-based threshold was applied; visual tracer uptake in dominant tumour lesions was required to be higher than the physiological liver background. The Retrospective inclusion into the analysis was systematically performed by reviewing all patients during the study period. That means patients included in this study: (1) showed a progression under prior PSMA-targeted radioligand therapy, confirmed by PSA increase > 25% and radiological progression on [⁶⁸Ga]Ga-PSMA-11 PET/CT; (2) were in ECOG performance status ≤ 3; and (3) received augmentation with a single-dose docetaxel chemotherapy.

The interval between docetaxel administration and [¹⁷⁷Lu]Lu-PSMA-617 administration ranged from 3 to 13 days, with a median of 7 days and a mean of 7.6 days. Eight out of ten patients received docetaxel prior to radioligand therapy, and two out of ten patients received docetaxel immediately after administration of [¹⁷⁷Lu]Lu-PSMA-617 and discharge from the therapy department. The mean administered activity of [^177^Lu]Lu-PSMA-617 was 6.6 ± 1.6 GBq (range: 3.2–8.2 GBq).

All patients were heavily pretreated, defined as having received a minimum of 4 prior systemic therapeutic lines for mCRPC: 100% (10/10) had received docetaxel chemotherapy, 50% (5/10) both docetaxel and cabazitaxel. 80% (8/10) had received at least one androgen receptor-directed agent (abiraterone or enzalutamide) prior to [^177^Lu]Lu-PSMA-617 RLT. Additionally, 30% (3/10) had undergone prior treatment with Radium-223. All patients had baseline [^68^Ga]Ga-PSMA-11 PET/CT before docetaxel-augmented RLT and restaging PET/CT 4–8 weeks later. Detailed information addressing patient characteristics and prior therapies are summarized in Table 1.

Patients receiving this off-label therapy underwent individual compassionate use treatments based on the German Pharmaceutical Act § 13 (2b) following interdisciplinary tumor board decisions. The Decision of a single-dose docetaxel regimen (50 mg/m²) was chosen to balance potential radiosensitizing effects with safety concerns. Given that all patients were heavily pretreated with most having already received full courses of docetaxel and/or cabazitaxel, the administration of a single dose allowed for a feasible and ethically acceptable approach in this end-stage population.

Patients were in detail informed about the highly experimental character and about the risks and potential side effects of this intervention and gave written informed consent. Additionally, patients consented to publication of any resulting data in accordance with the Declaration of Helsinki.

The analysis was approved by the local institutional review board (ethics committee permission number 140/17). [^177^Lu]Lu-PSMA-617 was administered during an inpatient stay in accordance with German radiation protection regulations. The mean administered activity of [^177^Lu]Lu-PSMA-617 was 6.6 ± 1.6 GBq (range: 3.2–8.2 GBq). To reduce side effects, each patient received intravenous hydration (1000 mL 0.9% NaCl) 30 min before to 120 min after injection of the radioligand as well as cooling of the salivary glands.

**Table 1:** Patient and treatment characteristics

Characteristics (*n* = 10)	Value
Age, years	
Median,	68 (51–74)
Age ≥ 70	40% (4)
ECOG performance score category	
≤ 1	80% (8)
> 1	20% (2)
PSA at start of therapy, ng/mL	
Median PSA	317
> 1000	10% (1)
Prior therapies	
Surgery	60% (6)
Radiation therapy	40% (4)
Abiraterone or enzalutamide	80% (8)
Abiraterone + enzalutamide	20% (2)
Docetaxel or cabazitaxel	100% (10)
Docetaxel + cabazitaxel	50% (5)
^223^Ra	30% (3)
Prior [^177^Lu]Lu-PSMA-617	100% (10)
Median cycles	5 (1–10)
Median cumulative activity, GBq	27,5 (7,5–77,6)
Prior [^225^Ac]Ac-PSMA-617	20% (2)
Median cycles	2 (1–3)
Median cumulative activity, MBq	10,3 (5,7–14,8)
Site of metastases before therapy, % (n)	
Any bone	100% (10)
Predominantly bone	80% (8)
Lymph node	80% (8)
Liver	10% (1)
Lung	10% (1)

ECOG: Eastern Cooperative Oncology Group, GBq: Gigabequerel, MBq: Megabequerel, PSA: prostate-specific antigen; PSMA: prostate-specific membrane antigen

### Docetaxel chemotherapy

The applied dosage of docetaxel was 50 mg/m^2^. The chemotherapy was administered within one week to the administration of [^177^Lu]Lu-PSMA-617. The application was performed by the patient’s uro-oncologist. In one case, the dosage was reduced to 35 mg/m^2^ by the oncologist.

### Response and toxicity assessment

Response to treatment was evaluated based on the change of serum PSA level. Serum PSA level was determined prior to and 4–8 weeks after therapy. Biochemical partial response (PR) was defined as a ≥ 50% decrease, and biochemical stable disease (SD), as an intermediate change (< 50% decrease to < 25% increase). Biochemical progressive disease (PD) was defined using Prostate Cancer Clinical Trials Working Group 3 criteria as a relative PSA increase of ≥ 25% and an absolute increase of at least 2 ng/mL [12]. Serum PSA level was determined prior to and 4–8 weeks after docetaxel-augmented RLT.

Molecular imaging-based response was assessed by calculating the whole-body total lesion PSMA (TLP) and molecular tumor volume (MTV) derived from [^68^Ga]Ga-PSMA-11 PET/CT images, taken before and after augmentation therapy approach by applying a semi-automatic tumor segmentation using Syngo.Via (Enterprise VB 30, Siemens, Erlangen, Germany). The PET Response Criteria in Solid Tumors (PERCIST) version 1.0 [13] were slightly modified: PR was defined as a reduction in TLP or MTV > 30%, PD as an increase exceeding 30% or the appearance of new metastases, and SD as a change ranging between − 30% and + 30%. To evaluate safety, blood counts and function of kidney and liver routine laboratory tests were performed at baseline and 4–8 weeks after docetaxel-augmented RLT. Toxicity was recorded using the Common Terminology Criteria for Adverse Events (CTCAE) version 5.0 [14]. Xerostomia was evaluated on patient reports via a questionnaire during hospitalization and at each outpatient visits. The used questionnaire was developed by our department and was based on CTCAE including dry mouth feeling during the day, at night or while eating, swallowing problems and intake alterations.

### Statistical analysis

Data on patients, treatment, and response characteristics are presented as descriptive statistics where applicable. PSA-based progression-free survival (PSA-PFS) and overall survival (OS) were analyzed using Kaplan–Meier statistics. PSA-PFS was defined as the time from the administration of docetaxel-augmented RLT until the event of either (1) documented biochemical PD; (2) death from any cause or (3) the latest study visit. OS was defined as the interval from the docetaxel-augmented RLT to the occurrence of either (1) death from any cause or (2) the latest study visit. The level of significance was defined as *p* < 0.05. SPSS version 29 software (SPSS Inc., Chicago, IL, USA) and Prism 9 (GraphPad Software, San Diego, USA) was used for all statistical analysis.

## Results

We retrospectively analyzed outcome and safety of single-dose docetaxel augmented [^177^Lu]Lu-PSMA-617 RLT in 10 mCRPC patients with progression under [^177^Lu]Lu-PSMA-617 RLT or [^225^Ac]Ac-PSMA-617 / [^177^Lu]Lu-PSMA-617 tandem RLT. The population was comprised by late-stage/end-stage and heavily pretreated patients as presented in Table 1. On average 5 cycles (range: 1–10) of [^177^Lu]Lu-PSMA-617 with a median activity of 27.5 GBq were administered before starting docetaxel-augmented RLT. Two patients had already received [^225^Ac]Ac-PSMA-617 / [^177^Lu]Lu-PSMA-617 tandem RLT with an average of 2 cycles and median activity of 10 MBq [^225^Ac]Ac-PSMA-617.

### Therapeutic efficacy

#### Biochemical response rate

**Fig. 1:**
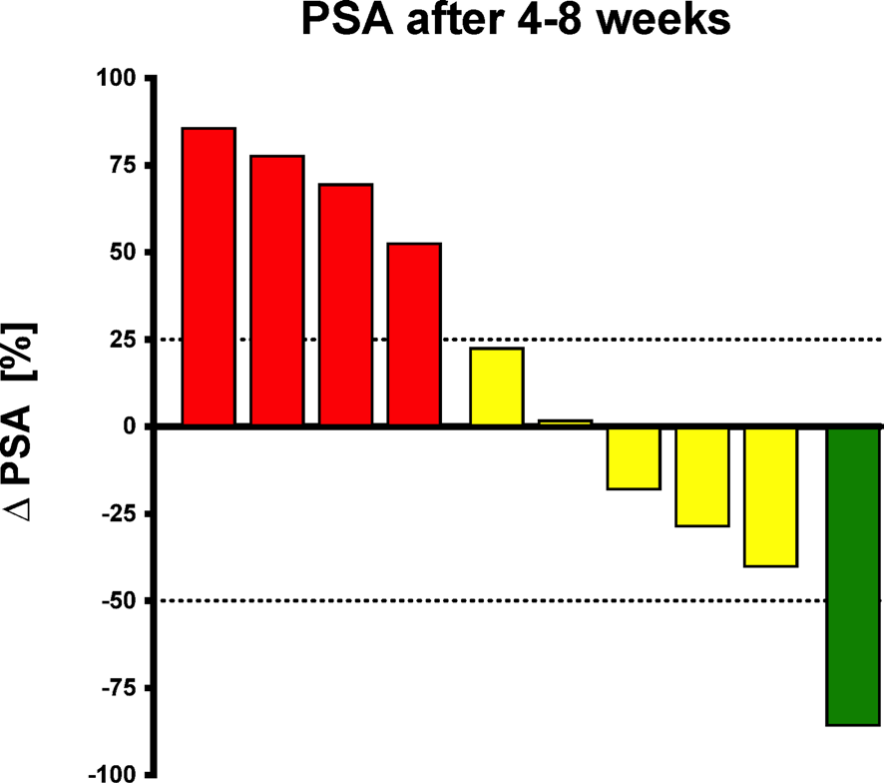
Relative changes of PSA in percent, 4–8 weeks after augmentation therapy

PSA concentration had declined from baseline levels in 4/10 (40%) of the patients, with a PR (decrease > 50%) in one patient (10%). In total, 5/10 (50%) patients showed a stable disease. 4/10 (40%) patients had a biochemical progression (PSA increase of > 25%). Therefore, the objective response rate for biochemical response was 10% (95% CI: 0.3–44.5%). The disease control rate was 60% (95% CI: 26.2% – 87.8%). The relative changes of PSA are illustrated as a waterfall plot in Fig. 1. The detailed values of each patient are compiled in Table 2.

#### Molecular imaging response rate

Molecular imaging-based response was available for 8/10 (80%) patients. The response classification demonstrated a complete concordance between MTV and TLP for 8/8 (100%) Patients. PR was reached by 2/8 patients (25%) with a decrease of TLP and MTV of > 30%. 3/8 patients (37.5%) showed SD with a change of TLP and MTV between − 30% and + 30%. A progression (increase of TLP and MTV > 30%) was found in 3/8 (37.5%). The objective response rate for imaging was 25% (95% CI: 2.5–55.6%) and the disease control rate was 62.5% (95% CI: 24.5% – 91.5%).

**Fig. 2:**
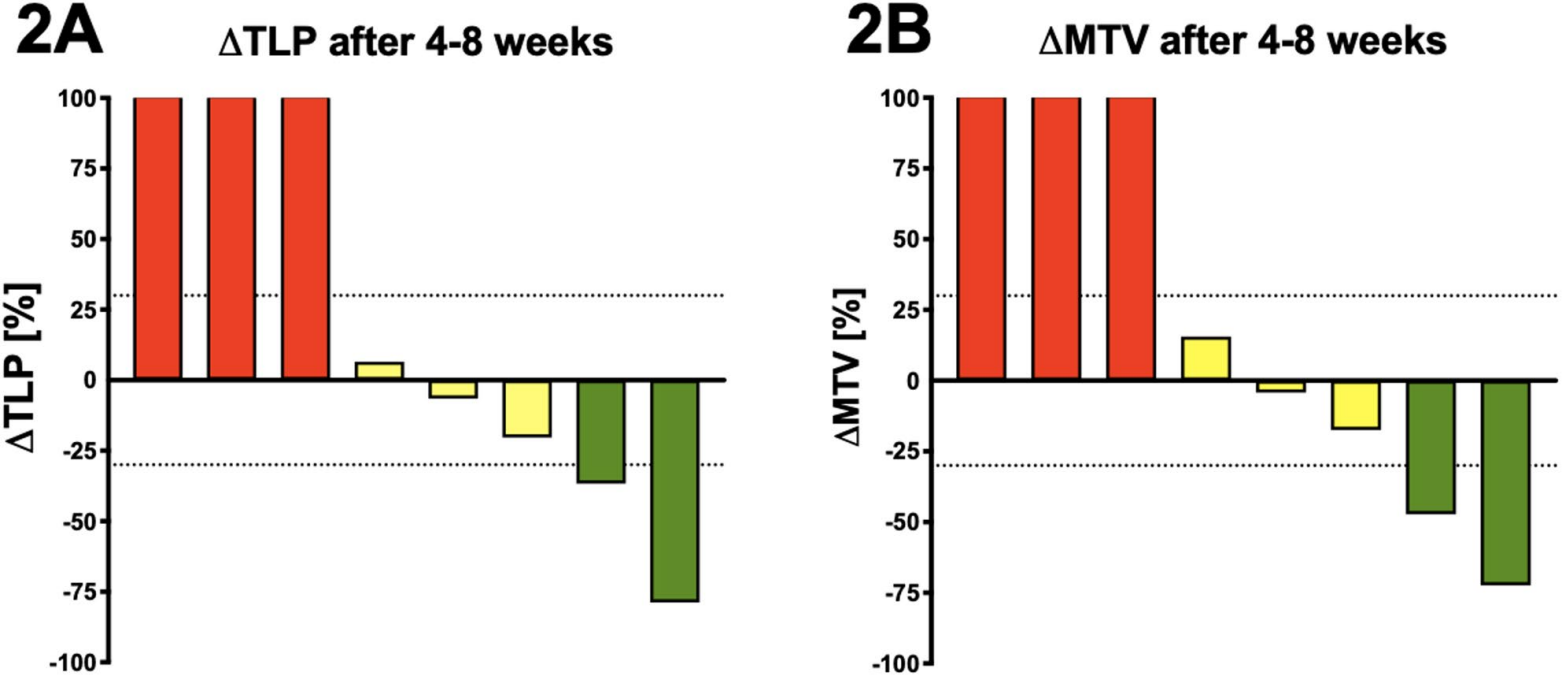
**A** and **B** Relative changes of TLP, MTV in percent. Columns were truncated at 100% to improve visualization

Comparing TLP/MTV to the PSA response, 4/8 patients (50%) showed a concordance. Two of the discordant patients had stable molecular imaging while one exhibited biochemical PR and the other showed biochemical PD based on the applied PSA criteria. One patient with PR and one patient with PD, assessed by molecular imaging presented biochemical SD. Waterfall plots of MTV and TLP are presented in Fig. 2A and 2B, respectively. Table 2

 Summarizes the molecular imaging-based response data. The relative changes of each parameter are illustrated in Table 3.

**Table 2:** Absolute values of TLP, MTV and PSA prior to and after single-dose docetaxel augmented [^177^Lu]Lu-PSMA-617 RLT (values were rounded to whole numbers)

Patient number	TLP prior therapy [ml*SUV]	MTV prior to therapy [ml]	TLP after therapy [ml*SUV]	MTV after therapy [ml]	PSA prior to therapy [ng/ml]	PSA after therapy [ng/ml]
**1**	3035	723	2417	597	582	81
**2**					241	296
**3**	8692	681	1843	188	45	32
**4**	2046	398	1913	381	274	276
**5**	2475	568	8000	1250	242	411
**6**	9194	1475	9793	1704	121	225
**7**	5993	1318	19,646	3726	645	1148
**8**	2329	345	11,031	1431	295	241
**9**					276	422
**10**	5690	967	3602	510	200	119

No imaging Data available for patients 2 and 9

**Table 3:** Relative changes in percentage of TLP, MTV and PSA

Patient number	∆TLP [%]	∆MTV [%]	∆PSA [%]	TLP Response	MTV Response	PSA Response
**1**	-20.3	-17.5	-86.1	SD	SD	PR
**2**			22,8			SD
**3**	-78.8	-72.4	-28.8	PR	PR	SD
**4**	-6.5	-4.2	0.7	SD	SD	SD
**5**	223.2	120.0	69.8	PD	PD	PD
**6**	6.5	15.6	86.0	SD	SD	PD
**7**	227.9	182.7	78.0	PD	PD	PD
**8**	373.6	315.0	-18.3	PD	PD	SD
**9**			52.9			PD
**10**	-57,96	-47,24	-40,50	PR	PR	SD

No imaging Data available for patients 2 and 9

### Survival data

**Fig. 3:**
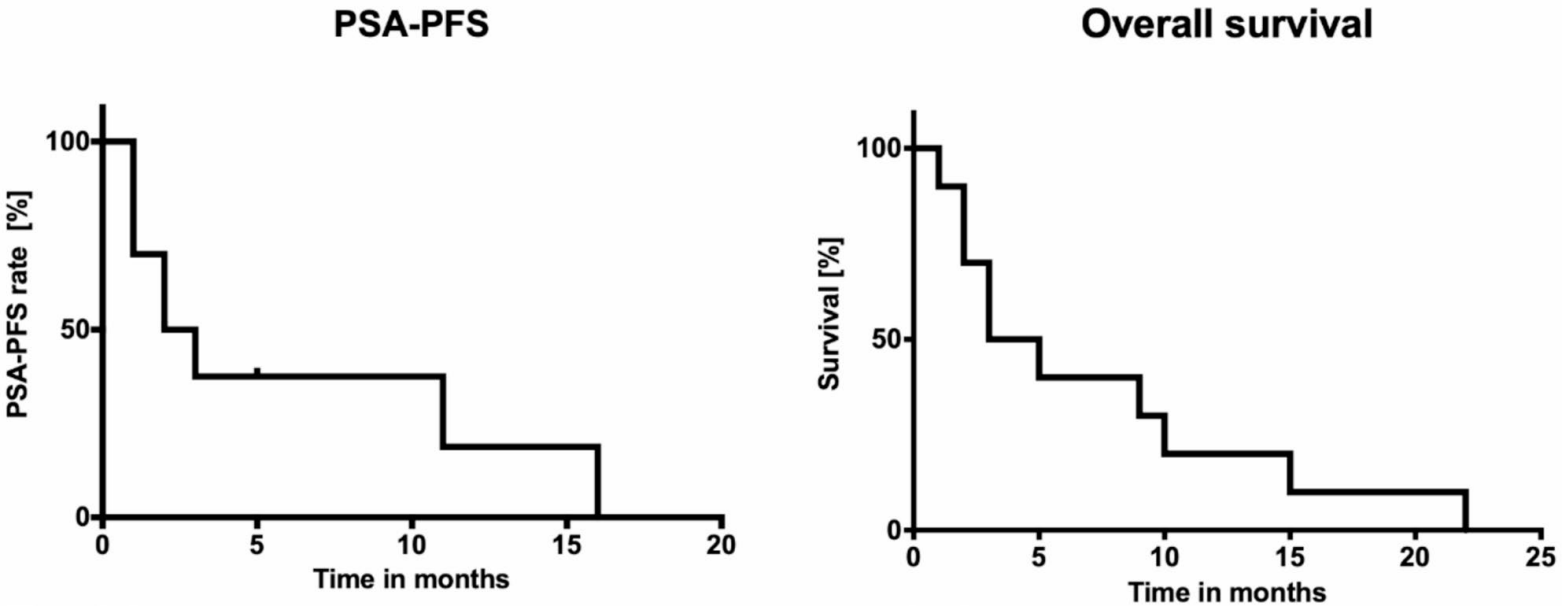
Median PSA-PFS and OS were 4.4 months (95%CI: 1.3–7.6 months) and 7.2 months (95%CI:2,9–11,5 months), respectively

After single-dose docetaxel augmented [^177^Lu]Lu-PSMA-617 RLT, patients continued to receive [^177^Lu]Lu-PSMA-617 RLT, 4 patients received [^225^Ac]Ac-PSMA-617 / [^177^Lu]Lu-PSMA-617 tandem-therapy later on (median 4.5 months after augmentation therapy). The median duration of follow-up was 6.0 months (range: 1–19 months). At the time of analysis all patients died due tumor related death. Median PSA-PFS was 4.4 months (95%CI: 1.3–7.6 months) and median OS was 7.2 months (95%CI: 2.9–11.5 months), **(**Fig. 3**)** calculated from the start of augmentation therapy.

### Toxicity

**Fig. 4:**
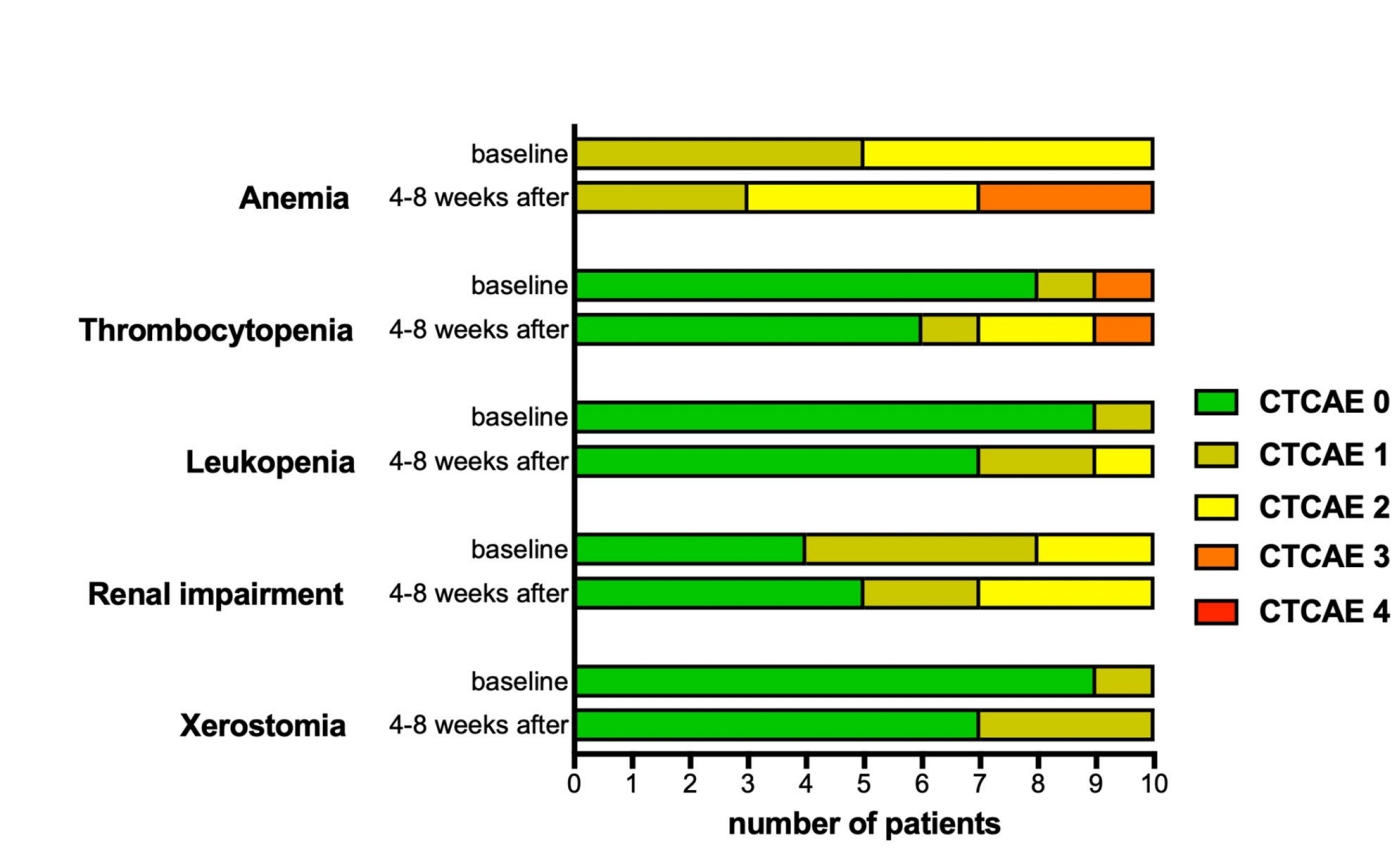
Bar diagram presenting adverse events categorized according to the ‘common terminology criteria for adverse events’ (CTCAE), each apportioned for baseline and 4–8 weeks after augmentation therapy ([^177^Lu]Lu-PSMA-617 RLT + single-dose docetaxel)

Docetaxel-augmented RLT was well-tolerated without any serious acute adverse events. Figure 4 presents the documented adverse events, based on CTCAE terminology, before (baseline) and 4–8 weeks after (follow-up) the augmentation therapy. During the first 4–8 weeks after augmentation therapy, three patients exhibited transient grade 3 anemia. Two of them had grade 2 anemia at baseline, the third one showed a grade 1 anemia at baseline. In addition, one transient grade 1 anemia occurred during the first 4–8 weeks.

Two patients developed a grade 2 thrombocytopenia (both presented a grade 0 at baseline). One case of transient leukopenia grade 2 was observed (baseline grade 0). One patient developed a grade 2 renal impairment (baseline grade 1). The renal function remained stable at grade 2 during the follow up and did not deteriorate.

## Discussion

This is the first study to evaluate the efficacy and safety of single-dose docetaxel augmented [^177^Lu]Lu-PSMA-617 RLT in patients with advanced mCRPC who have progressed on preceding PSMA-RLT. The analysis demonstrates that adding a single standard dose of docetaxel to [^177^Lu]Lu-PSMA-617 RLT did achieve biochemical disease stabilization in about two-third of these highly challenging late-stage/end-stage treatment-refractory patients for 4–8 weeks. PSA-PFS and OS thereafter were 4.4 months (95%CI: 1.3–7.6 months) and 7.2 months (95%CI: 2.9–11.5 months), respectively. Furthermore, docetaxel-augmented RLT led to either stabilization or decrease of viable tumor burden, measured as MTV/TLP on the [^68^Ga]Ga-PSMA-11 PET/CT in also in about two-third of the patients. The safety profile of this augmented RLT approach signaled feasibility without severe adverse events.

The observed efficacy of augmentation therapy may be attributed to the complementary mechanisms of action of taxane chemotherapy and [^177^Lu]Lu-PSMA-RLT. Taxanes inhibit cancer cell mitosis, while [^177^Lu]Lu-PSMA targets PSMA-positive cells, potentially leading to synergetic therapeutic effects. Another hypothesis is that chemotherapy could play a role as radiosensitizer for RLT enhancing the therapeutic effect of the beta-emitter. Our findings are consistent with recent studies suggesting that combination therapies may overcome resistance mechanisms associated with monotherapy [10, 15]. This concept, combining chemotherapy and RLT has been examined previously in another tumor entity, namely in patients with neuroendocrine tumors treated with [^177^Lu]Lu-DOTATAE and chemotherapy (temozolomide-capecitabine) [16, 17]. In this context, this combination therapy showed promising results improving survival (PFS and OS) with acceptable side-effect profile, similarly to the presented results on mCRPC.

Recently, Azad et al. have presented the initial data of the “UpFront-PSMA” trial comparing sequential [^177^Lu]Lu-PSMA-617 and docetaxel versus docetaxel alone in patients with metastatic hormone-sensitive prostate cancer [18]. In this multicenter, open-label, randomized, phase II study, 25/61 (41%) patients of the [^177^Lu]Lu-PSMA-617 plus docetaxel group had undetectable PSA at 48 weeks compared with 10/61 (16%) patients in the docetaxel alone group (OR 3·88, 95% CI 1·61–9·38; *p* = 0·0020). Severe adverse events occurred in both groups: 16/63 (25%) patients in the [^177^Lu]Lu-PSMA-617 plus docetaxel group (none were definitely related to [^177^Lu]Lu-PSMA-617 RLT) and 16/63 patients (25%) in the docetaxel alone group. Therefore, the authors concluded that [^177^Lu]Lu-PSMA-617 RLT followed by docetaxel improved antitumor activity in patients with de-novo high-volume metastatic hormone-sensitive prostate cancer compared with docetaxel alone, without increased toxicity. Comparison between the cited report and the results presented in this analysis is not applicable without restrictions due to the differing patient characteristics including the divergent course of disease (mCRPC in our study vs. metastatic hormone-sensitive prostate cancer in the UpFront-PSMA trail) were analyzed. However, both studies showed promising results of combining therapies ([^177^Lu]Lu-PSMA-617 RLT+ docetaxel) in patients with prostate cancer.

In our previous pilot study using a combination therapy, although a single-cycle of tandem therapy (low activity of ^225^Ac + standard activity of ^177^Lu) a stabilization of the disease was achieved in 90% of mCRPC patients who showed insufficient response to [^177^Lu]Lu-PSMA monotherapy, leading to PFS of 19 weeks (4,7 months) and OS of 48 weeks (12 months) [9]. The results of this study are to some extent consistent with the results of our earlier pilot study, although the slightly lower efficacy may be explained by the differing patient cohorts (patients with inadequate response to LuPSMA in the tandem RLT paper vs. patients with biochemical and molecular imaging progression under LuPSMA in the current study).

In a Phase I, dose-escalation study of 15 men with mCRPC, 177Lu-J591 was combined with docetaxel (2 cycles of docetaxel, followed by 2 cycles of ^177^Lu-J591, augmented by one cycle of docetaxel, and docetaxel every 3 weeks on an ongoing basis) [19]. This study showed a biochemical response (PSA50) in 73% of patients and a response according to the RECIST criteria in 60% of patients. The combination approach was well tolerated, and no dose-limiting toxicity was detected, providing some early support for this approach. However, our results are only comparable to a limited extent to this phase I study, as their patients were ^177^Lu-PSMA-naïve and the protocol differs significantly from our study protocol which results in different accumulation and distribution kinetics comparing to the small-molecule ligand PSMA-617. Nonetheless, both studies provide results supporting the use of this augmented radioigand therapy in mCRPC.

The single-dose docetaxel augmented [^177^Lu]Lu-PSMA-617 radioligand therapy was safe and generally well-tolerated. The incidence of severe adverse events was low and most adverse effects were manageable with supportive care. The safety of combining the two modalities appears to be acceptable with adverse events consistent with those reported for each therapy individually. The most common adverse events were hematotoxicity. Grade 3 anemia occurred in 3/10 (30%) patients 4–8 weeks after combination therapy. This is higher compared to the toxicity data of the [^177^Lu]Lu-PSMA trials such Vision and TheraP trial which recorded a grade 3 anemia in 12.9% and in 8% of patients [2, 4].

Regarding other hematotoxicities, no grade 3/4 thrombocytopenia or leukopenia were observed during the 4–8 weeks follow-up. No grade 3/4 nephrotoxicity was noted in any patient, only one incident of transient grade 2 renal impairment occurred 3 weeks after augmentation therapy. This patient recovered and maintained an estimated-GFR of 80 mL/min until death due to tumor progression. It is relevant to note that our patients were pretreated with [^177^Lu]Lu-PSMA radioligand therapy. Therefore, some patients had already developed xerostomia at baseline which stayed throughout combination therapy. Only one patient (10%) developed a new xerostomia grade 1 two weeks after augmentation therapy. Compared to the [^177^Lu]Lu-PSMA trials [2, 3] we observed lower xerostomia rates after the combination therapy, despite the previous cycles of [^177^Lu]Lu-PSMA RLT (median 5, range: 1–10 cycles). UpFrontPSMA trial reported serious adverse events in 25% of patients in the [^177^Lu]Lu-PSMA RLT plus docetaxel group, none of which were clearly attributable to [^177^Lu]Lu-PSMA RLT. The same rate of 25% was observed in the docetaxel-alone group. In our study, the rate of serious adverse events was lower, possibly due to the use of only one single dose of docetaxel and one cycle of [^177^Lu]Lu-PSMA RLT.

The PSMAfore trial recently established [177Lu]Lu−PSMA − 617 as a superior option compared to a simple ARPI change in taxane-naive patients [20]. This move to earlier lines suggests that we should focus more on synergistic combinations rather than just sequential monotherapy. Current research such as the LuPARP trial showed that inhibiting DNA repair with Olaparib can ‘prime’ cells for radiation, achieving a 62% PSA50 response rate [21]. Similarly, dual radionuclide strategies using [161 Tb]Tb−PSMA-617 and [177Lu]Lu−PSMA − 617 are being explored to overcome resistance [22]. Our approach of augmenting RLT with a single dose of docetaxel follows this same logic. It is an attempt to intensify treatment to overcome resistance with an acceptable safety profile, especially for late-stage patients where effective options are increasingly limited.

### Limitation

Our analysis benefits from real world clinical data. However, there are limitations, including the retrospective design of a unicentric, small sample and the lack of a control group. A further limitation is the heterogeneity of included patients who were heavily pre-treated with different therapies. The cumulative activity of ^177^Lu before docetaxel-augmented RLT ranged between 7.5 and 77.6 GBq, which potentially presents a bias. Due to the heterogeneous population, there were no fixed activity protocols, but an individualized approach taking into account characteristics such as patient condition, previous response and tumor burden. Another limitation was the lack of a predefined, standardized interval between chemotherapy and PSMA-RLT. Regarding the potential influence of treatment timing on outcomes, the small sample size (*n* = 10) and variability in treatment sequencing prevented meaningful subgroup analysis or firm conclusions. Furthermore, OS and PFS data need to be interpreted with caution because patients received [^177^Lu]Lu-PSMA monotherapy as maintenance and 4 patients received [^225^Ac]Ac-PSMA / [^177^Lu]Lu-PSMA tandem therapy later. These factors impact generalizability of our results.

## Conclusion

This pilot experience demonstrates that adding one single-dose docetaxel application to [^177^Lu]Lu-PSMA radioligand therapy is feasible and may present an option for late-stage heavily pretreated mCRPC progressing on PSMA-RLT. Furthermore, investigation of earlier combination of the two modalities, radioligand therapy and taxane chemotherapy, might be supported by this very preliminary data. The findings warrant further exploration through clinical trials and potential consideration in future treatment protocols.

## Data Availability

The datasets generated during and analyzed during the current study are available from the corresponding author on reasonable request.
